# Noninvasive Monitoring of Inflammatory Processes by Myeloid Cell–Directed PET Tracers in an Experimental Severe Acute Respiratory Syndrome Coronavirus 2 Infection Model

**DOI:** 10.2967/jnumed.125.269721

**Published:** 2026-01

**Authors:** Marieke A. Stammes, Gerrit Koopman, Teresa R. Wagner, Bjoern Traenkle, Philipp D. Kaiser, Petra Mooij, Nicole van der Werff, Roja Fidel Acar, Kinga P. Böszörményi, Simone Blaess, Stefania Pezzana, Gerald Reischl, Andreas Maurer, Jan A.M. Langermans, Ulrich Rothbauer, Manfred Kneilling, Dominik Sonanini

**Affiliations:** 1 Biomedical Primate Research Centre, Rijswijk, The Netherlands;; 2 NMI Natural and Medical Sciences Institute, University of Tübingen, Reutlingen, Germany;; 3 Werner Siemens Imaging Center, Department of Preclinical Imaging and Radiopharmacy, University Hospital Tübingen, Eberhard Karls University Tübingen, Tübingen, Germany;; 4 Cluster of Excellence iFIT 2180 “Image-Guided and Functionally Instructed Tumor Therapies,” Eberhard Karls University, Tübingen, Germany;; 5 Department of Population Health Sciences, Unit Animals in Science and Society, Faculty of Veterinary Medicine, Utrecht University, Utrecht, The Netherlands;; 6 Pharmaceutical Biotechnology, Eberhard Karls University Tübingen, Tübingen, Germany;; 7 Department of Dermatology, Eberhard Karls University, Tübingen, Germany; and; 8 Department of Medical Oncology and Pneumology (Internal Medicine VIII), Eberhard Karls University, Tübingen, Germany

**Keywords:** SIRPα, TSPO, nanobody, PET/CT, COVID-19, monitoring of infection

## Abstract

Monitoring the presence and distribution of distinct immune cell populations is key in deciphering immunopathologic disease mechanisms. Considering the crucial role of myeloid cells in provoking hyperinflammatory responses associated with coronavirus disease 2019, a camelid-derived single-domain antibody specifically recognizing human signal-regulatory protein-α (SIRPα) as a biomarker for myeloid cells has been generated and radiolabeled with ^64^Cu, that is, [^64^Cu]copper-SIRPα-nanobody ([^64^Cu]Cu-SIRPα-Nb), for in vivo PET imaging. In this study, this PET tracer was used and validated to monitor the temporal dynamics of inflammatory processes during severe acute respiratory syndrome coronavirus 2 (SARS-CoV-2) infection in nonhuman primates. For further validation, the signal of [^64^Cu]Cu-SIRPα-Nb was compared with the signal of TSPO-targeting [^18^F]fluorine-*N*,*N*-diethyl-2-(2-(4-(2-fluoroethoxy)phenyl)-5,7-dimethylpyrazolo[1,5a]pyrimidin-3-yl)acetamide ([^18^F]F-DPA714). **Methods:** Six cynomolgus macaques (*Macaca fascicularis*) were experimentally infected with SARS-CoV-2 and subsequently monitored for 7 wk. We performed a baseline [^64^Cu]Cu-SIRPα-Nb PET/CT before infection, followed by repetitive PET/CT on days 3, 10, 24, and 52 after infection covering the head, thorax, and abdominal region. Of the 6 macaques, 3 underwent additional TSPO-targeting [^18^F]F-DPA714 PET/CT investigations at baseline and at days 2, 9, 23, and 51 after infection. Tracheal and nasal swabs were taken to monitor the course of infection, and changes in blood count and inflammatory mediators in the periphery blood were monitored. **Results:** Detection of subgenomic SARS-CoV-2 messenger RNA from tracheal and nasal swab samples confirmed viral infection of all animals. CT scans identified initial pulmonary lesions 2 days after infection. [^64^Cu]Cu-SIRPα-Nb PET/CT revealed increased tracer uptake in the mediastinal lymph nodes of all animals, but not in the lung lesions, 3 days after infection. [^18^F]F-DPA714 PET/CT showed increased uptake in both anatomically affected and unaffected lung tissues. The increased [^64^Cu]Cu-SIRPα-Nb uptake in the spleen and bone marrow of all animals was more pronounced 3 and 7 wk after infection. We detected individual differences in [^64^Cu]Cu-SIRPα-Nb uptake within the bone marrow and spleen, whereas [^18^F]F-DPA714 exhibited rather homogeneous uptake patterns. **Conclusion:** Our findings indicate that [^64^Cu]Cu-SIRPα-Nb is a versatile tool for quantitative monitoring of whole-body distribution, temporal distribution, and accumulation dynamics of myeloid cell subpopulations even in moderately inflamed tissues and affected lymphatic organs during SARS-CoV-2 infection.

## Visual Abstract

**Figure:**
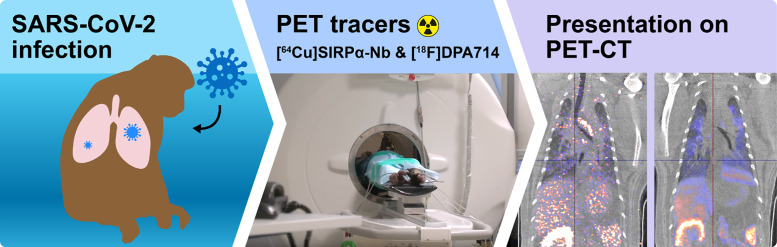

Since the beginning of the coronavirus disease 2019 (COVID-19) pandemic, researchers have gained extensive insights into the course of the disease and the pathologic mechanisms of severe acute respiratory syndrome coronavirus 2 (SARS-CoV-2) (1,2*)*. After an initial phase of viremia, some patients acquire uncontrolled lung inflammation (3,4*)*, whereas others experience continuation of symptoms months after the initial infection (5,6*)*. However, because reliable biomarkers are still missing, clinicians are unable to predict disease progression.

There is increasing evidence that myeloid cells play a decisive role in the development of COVID-19–associated acute respiratory distress syndrome and severe disease progression (1,2,7,8*)*. Myeloid cells also appear to be involved in the development of diffuse and long-lasting symptoms after disease convalescence (long COVID). In this context, SARS-CoV-2 infection was described as generating an inflammatory imprint in the monocyte or macrophage compartment and thus might alter macrophage effector functions, resulting in long-term immune aberrations in patients recovering from mild COVID-19 (9*)* and COVID-19–associated lung fibrosis (10*)*. These data suggest that local and systemic alterations in myeloid cell density, differentiation, and activation are drivers of COVID-19 severity and long COVID symptoms (8*)*, providing a clear rationale for detailed spatiotemporal investigations of myeloid cells.

Noninvasive PET/CT imaging offers a diagnostic approach to monitoring the spatiotemporal distribution of myeloid cells in vivo. In a recent [^18^F]fluorine-*N*,*N*-diethyl-2-(2-(4-(2-fluoroethoxy)phenyl)-5,7-dimethylpyrazolo[1,5a]pyrimidin-3-yl)acetamide ([^18^F]F-DPA714) PET/CT study of nonhuman primates (NHPs) infected with SARS-CoV-2, we determined a persistent increase in immune cell activation in the lungs and brain and detected TSPO expression, as a measure for macrophage activation, until day 30 after infection (11*)*. In addition, a first-in-human [^18^F]F-DPA714 PET/CT study yielded in vivo evidence of widespread neuroinflammation, which implies that long-term inflammation is among the pathophysiology hallmarks of COVID-19 (12*)*.

However, to better assess the impact of macrophages in COVID-19 disease pathogenesis, their diverse subpopulations need to be understood in terms of their proinflammatory and antiinflammatory properties and their role in resolving inflammation (13*)*. Thus, biomarkers indicative for myeloid cells are urgently needed. Human signal-regulatory protein-α (SIRPα) is highly and constitutively expressed on cells of the myelomonocytic lineage, particularly macrophages, monocytes, dendritic cells, and granulocytes, as well as on activated natural killer cells and neurons. Thus, SIRPα is highly expressed in myeloid cell–enriched organs such as the spleen, liver, lymph nodes (LNs), bone marrow, brain, and salivary glands (14,15*)*, whereas in the lungs, SIRPα expression is limited (16,17*)*. In addition, SIRPα functions as an inhibitor of phagocytosis when it binds to its ligand CD47 on target cells (18*)*. SIRPα-mediated inhibition of phagocytosis was shown to inhibit replication of viruses, including SARS-CoV-2, that use trafficking to the low pH endosomal compartment (19*)*. Preclinical mouse studies indicate that SIRPα overexpression leads to antiinflammatory macrophage polarization, whereas SIRPα blockade provokes a proinflammatory macrophage phenotype (20,21*)*. To address SIRPα as a biomarker for myeloid cells, we recently developed a set of human SIRPα–specific nanobodies (22*)*. Validation of a radiolabeled lead candidate, further referred as [^64^Cu]copper-SIRPα-nanobody ([^64^Cu]Cu-SIRPα-Nb), demonstrated its suitability for monitoring the dynamics of myeloid cells in a preclinical tumor mouse model (22*)*. [^64^Cu]Cu-SIRPα-Nb PET uptake increased in human SIRPα or human CD47 knock-in mice in various myeloid cell–enriched organs, such as the spleen, the tumor, and the salivary glands, compared with the uptake in wild-type animals or a nonspecific control tracer.

Here, we have extended the application of this tracer for quantitative high-resolution immuno-PET of myeloid cells in NHPs to elucidate their role in the development of inflammatory processes after exposure to SARS-CoV-2. In this study, we comparatively monitored the biodistribution and accumulation sites of myeloid cells in SARS-CoV-2–infected NHPs using our [^64^Cu]Cu-SIRPα-Nb and TSPO-targeting [^18^F]F-DPA714. This allowed us to uncover tracer-specific differences and gain deeper insights into the role of myeloid cells, in particular of macrophages in the pathogenesis of COVID-19.

## MATERIALS AND METHODS

### Ethics and Animals

This study was performed in 6 male cynomolgus macaques (*Macaca fascicularis*) at the Biomedical Primate Research Center under project license AVD5020020209404, which was issued by a competent national authority (Central Committee for Animal Experiments). The animals were socially housed, in pairs, during the study. All possible precautions were taken to ensure the welfare of and to avoid discomfort to the animals.

### Procedures

**FIGURE 1.**
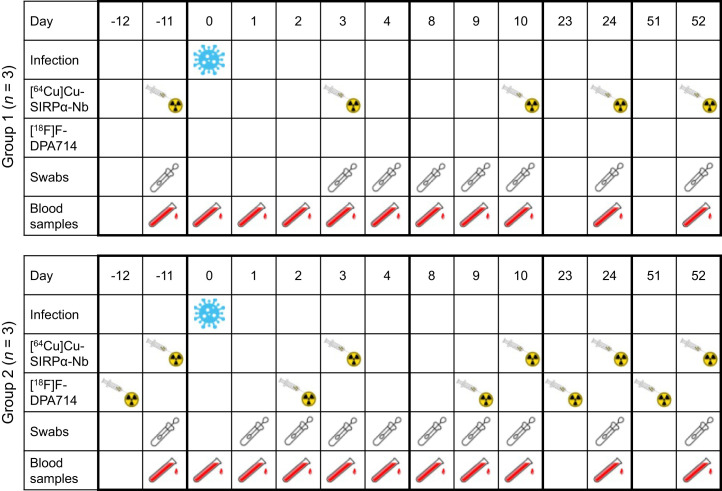
Timeline of study, including all experimental procedures performed.

All experimental procedures are described in detail in the supplemental materials (supplemental materials are available at http://jnm.snmjournals.org). Briefly, 6 animals were inoculated with a 50% tissue culture infectious dose of 1 × 10^5^ SARS-CoV-2 strain hCoV-19/Netherlands/NH-RIVM-27142/2021 (δ-variant, lineage B.1.617.2) via a combined intranasal and intratracheal route and divided over 2 groups of 3 animals. Group 1 received injections solely with [^64^Cu]Cu-SIRPα-Nb, whereas group 2 received injections with [^64^Cu]SIRPα-Nb and with [^18^F]F-DPA714 1 d on the forehand. All experimental interventions (intratracheal and intranasal infection, swab collections, blood sampling, and PET/CT) were performed under anesthesia (Fig. 1).

## RESULTS

### Binding Characteristics of SIRPα-Nb

**FIGURE 2.**
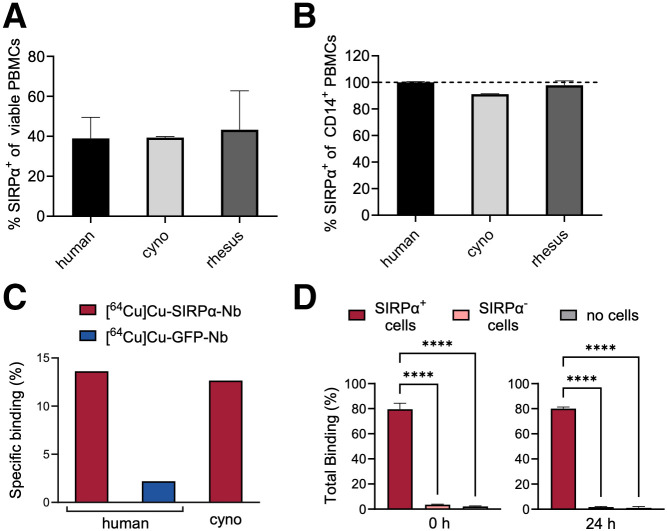
In vitro binding and cross-species reactivity of SIRPα-Nb. (A and B) Flow cytometry analysis of human (*n* = 3), cynomolgus (cyno; *n* = 1), and rhesus macaque (*n* = 1) peripheral blood mononuclear cells (PBMCs) with fluorescently labeled SIRPα-Nb of viable (A) and CD14+ (B) monocytes (technical triplicates each). (C) Specific binding of ^64^Cu-SIRPα-Nb to NHP and human PBMCs compared with unrelated control Nb ([^64^Cu]Cu-GFP-Nb). (D) Immunoreactivity of [^64^Cu]Cu-SIRPα-Nb using SIRPα-HT1080 cells and wild-type HT1080 cells directly after radiolabeling (0 h) and after 24-h storage in phosphate-buffered saline at room temperature. ****P < 0.0001.

Alignment of the amino acid sequences of human, cynomolgus, and rhesus SIRPα showed strong similarities in domain 2, representing the binding site of SIRPα-Nb (Supplemental Fig. 1A) (22*)*. In vitro cellular imaging showed that SIRPα-Nb recognized all tested SIRP variants except for cSIRPβ2 (Supplemental Fig. 1B). Flow cytometric analysis of peripheral blood mononuclear cells confirmed consistent binding of SIRPα-Nb across species, with more than 90% positive staining of the CD14-positive monocyte or macrophage population (Figs. 2A and 2B).

After site-specific conjugation with the metal chelator NODAGA and radiolabeling, we validated the specific binding of [^64^Cu]Cu-SIRPα-Nb to cynomolgus peripheral blood mononuclear cells in vitro. In accordance with the results from flow cytometric analysis, we detected similar binding for both species (Fig. 2B). [^64^Cu]Cu-SIRPα-Nb remained stable (Fig. 2C) and retained high immunoreactivity over 24 h at room temperature (Fig. 2D), allowing overnight shipment without quality loss. Furthermore, we confirmed specific in vivo binding of [^64^Cu]Cu-SIRPα-Nb by quantification of the SUV_mean_ in both salivary gland (high SIRPα expression) and muscle (low SIRPα expression) in the PET/CT before infection. The PET/CT with [^64^Cu]Cu-SIRPα-Nb (Supplemental Fig. 2A) were compared with [^18^F]F-DPA714 PET/CT (Supplemental Fig. 2B) and [^18^F]F-FDG PET/CT (Supplemental Fig. 2C), revealing average salivary gland-to-muscle ratios of 8.8 and 9.4, respectively, for the myeloid cell–specific tracers and a low salivary gland-to-muscle ratio of 1.5 for [^18^F]F-FDG (Supplemental Fig. 2D).

### Virus Infection

**FIGURE 3.**
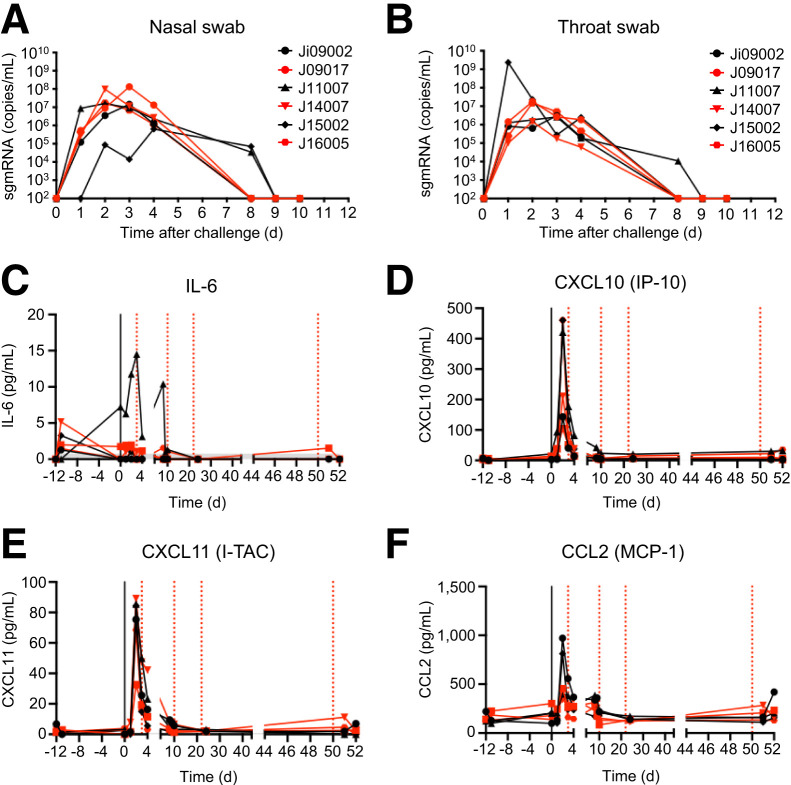
Virus load in nasal (A) and throat (B) samples, showing subgenomic SARS-CoV-2 RNA load (copies per milliliter) in SIRPα single–injected (red) or TSPO + SIRPα–injected (black) animals at different days after infection. (C–F) Proinflammatory cytokines and chemokines detected in plasma. Interleukin-6 (IL-6; C), CXCL10 (D), CXCL11 (E), and CCL2 (F) levels in pg/mL in SIRPα single–treated (red) or TSPO + SIRPα–treated (black) animals in time. Day 0 is time of infection (black vertical line). Time points of SIRP1α injection are indicated by vertical stippled red lines.

After exposure to SARS-CoV-2, we detected subgenomic viral messenger RNA in the tracheal and nasal swab samples of all 6 macaques from day 1 after infection up to 10 d after infection (Figs. 3A and 3B). Throughout the study, minor clinical signs presumably related to SARS-CoV-2 infection, i.e., an occasional cough, were observed in all animals, similar to what has been described in the literature (23*)*. However, in the serum of SARS-CoV-2–infected macaques, we found a transient virus infection–related increase in 2 interferon-γ–driven inflammatory cytokines, CXCL10 and CXCL11, and an increase of CCL2, whereas interleukin-6 was elevated in only 1 animal (Figs. 3C–3F). Other inflammatory cytokines, such as interferon-γ and CXCL8, did not reveal consistent changes associated with SARS-CoV-2 infection (Supplemental Fig. 3) or were entirely below the detection limit (e.g., tumor necrosis factor-α and interleukin-1β).

Overall, no global differences were observed between the 3 SARS-CoV-2–infected NHPs that underwent 5 [^64^Cu]Cu-SIRPα-Nb PET/CT and the 3 NHPs that received 5 additional [^18^F]DPA714 PET/CT investigations. Both [^64^Cu]Cu-SIRPα-Nb and [^18^F]F-DPA714 injections were well tolerated, with no observed clinical symptoms. However, a temporary reduction in lymphocytes (T and B cells), monocytes, and dendritic cells occurred in all animals after [^64^Cu]Cu-SIRPα-Nb injection (Supplemental Fig. 3).

### PET/CT

#### Lower Respiratory Tract

**FIGURE 4.**
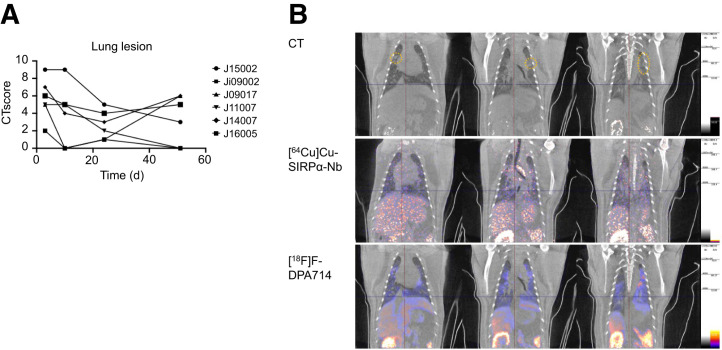
Lung lesions. (A) Semiquantitative scoring of lung lesions over time showing that in all animals, lesions were detected 1 or more days after infection. (B) Representative coronal CT and PET/CT slices of animal J15002 obtained at day 10 after infection ([^64^Cu]SIRPα-Nb) or day 9 after infection ([^18^F]DPA714) and showing several ground glass opacities, which are marked with orange stippled circles on CT.

After detection of viral infection in the respiratory tract, we performed CT to identify and monitor lesions in the lungs associated with SARS-CoV-2 infection (Fig. 4). Not all lung lesions were resolved by the end of the study, and upcoming lesions were identified over time. When scoring the severity of the SARS-CoV-2 infection, a maximum score of 9 of 30, mild-to-moderate lung inflammation was found in all SARS-CoV-2–exposed NHPs (Fig. 4A). The areas with enhanced [^64^Cu]Cu-SIRPα-Nb uptake in the lung did not correlate with the inflammatory lung lesions on CT. For [^18^F]F-DPA714, there was a correlation between lung lesions and areas of increased uptake (Fig. 4B).

We also examined the uptake of both tracers in anatomically unaffected lung tissue and found a negligible increase in [^64^Cu]Cu-SIRPα-Nb uptake, whereas in agreement with our recently published results (11*)*, an approximately 1.5-fold increase in the SUV_mean_ of [^18^F]F-DPA714 was observed (Supplemental Fig. 4).

#### Upper Respiratory Tract

##### Nasal Cavity

**FIGURE 5.**
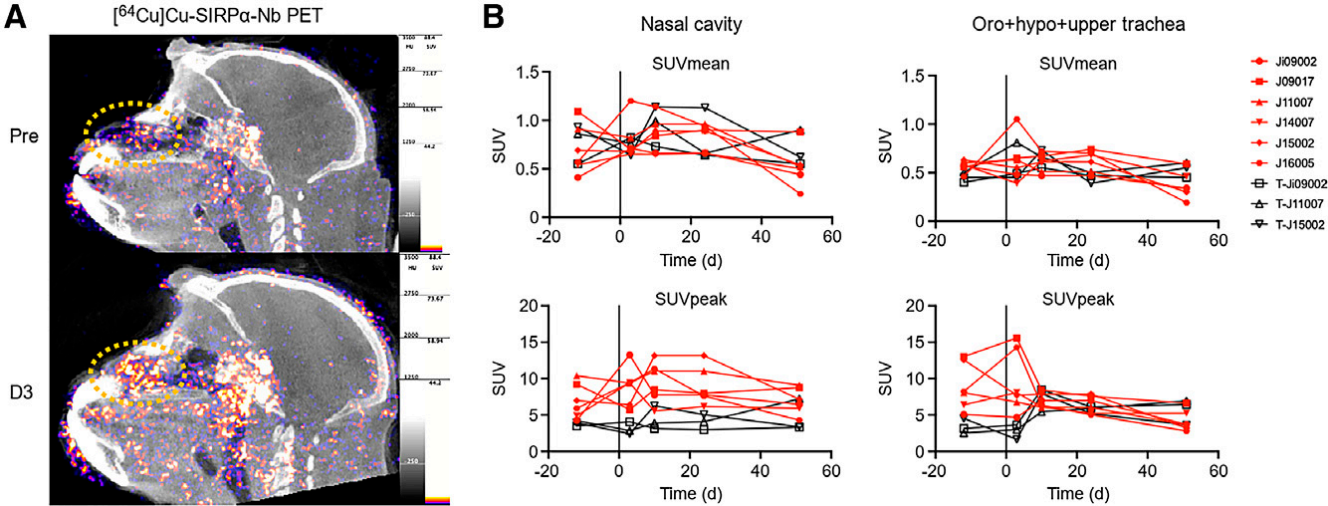
Visualization of nasal cavity and quantification of tracer signal in nasal cavity and upper respiratory tract. (A) Representative sagittal PET/CT slice showing increase in signal detected in nasal cavity from before infection (Pre) to day 3 (D3), which is concentrated on nose for [^64^Cu]SIRPα-Nb (marked with orange stippled circles). (B) Quantification of different regions of interest in nasal cavity and upper respiratory tract in SIRPα single–injected (red) or TSPO + SIRPα–injected (black) animals in time.

For the nasal cavity, we used a standardized sphere and anatomic boundaries to draw each region of interest. Before analyzing the data, we confirmed that the anatomic density of the region of interest defining the nasal cavity was comparable over time. [^64^Cu]Cu-SIRPα-Nb PET revealed an increased SUV_mean_ in the nasal cavity in all animals after infection (Fig. 5). This phenomenon was observed in 3 macaques on day 3 after infection and in 3 macaques on day 10 after infection (Fig. 5B). We still detected an elevated SUV_mean_ in the nasal cavity in 5 animals at day 24 after infection compared with before infection, although we were unable to detect subgenomic viral messenger RNA in the nasal swab 8–10 d after infection. However, these temporal differences were not associated with simultaneous upregulation of cytokines measured in the blood, indicating immune responses at the local tissue level (Fig. 5B).

##### Oropharynx, Hypopharynx, and Upper Trachea

For both tracers, we determined only minor alterations of the SUV_mean_ at the first time point after infection within the oropharynx, hypopharynx, and upper part of the trachea. The SUV_peak_ of the [^64^Cu]Cu-SIRPα-Nb scans showed relatively higher values before infection than did those of [^18^F]F-DPA714 (Fig. 5B). After infection, [^64^Cu]Cu-SIRPα-Nb uptake increased in 3 animals as early as 3 d after infection and decreased thereafter (Fig. 5B), whereas in the other 3 animals, [^64^Cu]Cu-SIRPα-Nb uptake decreased directly after infection. [^64^Cu]Cu-SIRPα-Nb uptake remained at identical levels between 3 and 10 d after infection. [^18^F]F-DPA714 exhibited the opposite uptake pattern, showing a low SUV_peak_ before infection that increased from day 3 until day 10 after infection (Fig. 5B).

#### Lymphatic Organs

Because SARS-CoV-2 infection is known to affect multiple systems in the body, including the lymphatic organs, we further analyzed [^64^Cu]Cu-SIRPα-Nb and [^18^F]F-DPA714 uptake patterns within the centrally located draining LNs of the lungs as central modulators of the disease, in the spleen, and in the bone marrow.

##### LNs

**FIGURE 6.**
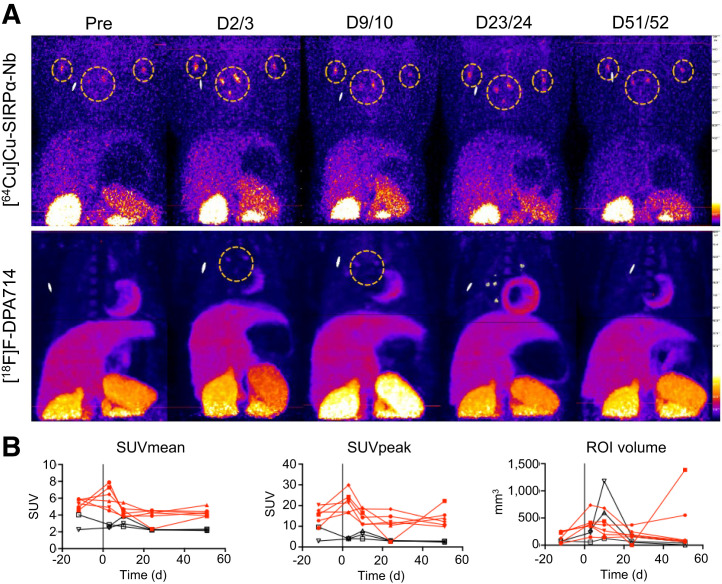
Visualization (A) and quantification (B) of mediastinal LNs, with both tracers followed over time in SIRPα single–injected (red) or TSPO + SIRPα–injected (black) animals. Orange stippled circles mark infection. D = day; hypo = hypopharynx; ortho = oropharynx; Pre = before infection; ROI = region of interest.

Mediastinal LNs were determined solely by the SUV threshold. We identified an increase in the [^64^Cu]Cu-SIRPα-Nb–associated SUV_mean_ and SUV_peak_ and an increase in active volume in the LNs for all SARS-CoV-2–infected animals (Fig. 6A). At baseline (before infection), we determined an average [^64^Cu]Cu-SIRPα-Nb PET–positive volume of 156 mm^3^ (range, 29–260 mm^3^), which increased to 403 mm^3^ (range, 148–737 mm^3^) 3 d after infection. It subsequently decreased with large individual differences until day 51 after infection (Fig. 6B). The correlation between SUV_mean_ and SUV_peak_ was positive for all time points (*r* = 0.51) and remained approximately the same over time, suggesting that the increased [^64^Cu]Cu-SIRPα-Nb PET–positive volume of the LNs was not affected by necrosis in the central parts. In addition to the increasing volume, we detected an average 1.44-fold increase (range, 1.11–1.60) in the SUV_mean_ in 3 of 6 animals on day 3 after infection (Fig. 6B). For [^18^F]F-DPA714, the visualization of the LNs was less clear and reflected in the SUV_mean_ and SUV_peak_, which stayed roughly the same over time (Fig. 6B).

##### Bone Marrow

**FIGURE 7.**
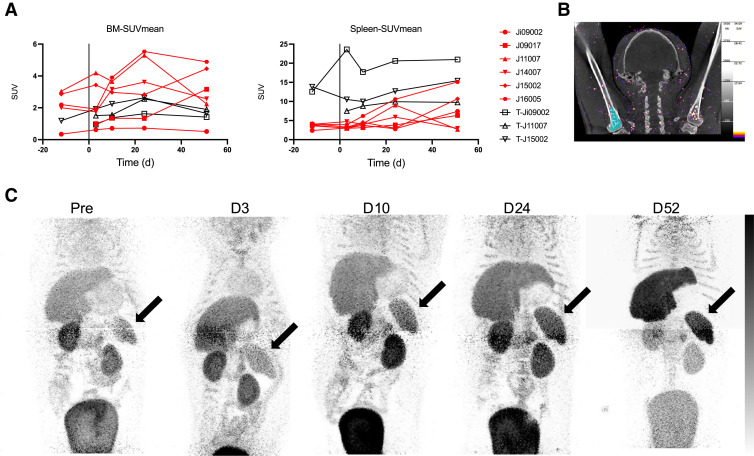
Quantification (A) of tracer signal in spleen and bone marrow (BM), as measured in left upper arm (B) in SIRPα single–injected (red) or TSPO + SIRPα–injected (black) animals. (C) Visualization of liver, kidney, and spleen (marked with arrow) uptake and clearance of [^64^Cu]SIRPα-Nb for all injections. D = day; Pre = before infection.

[^64^Cu]Cu-SIRPα-Nb PET/CT showed a clear increase in tracer uptake (SUV_mean_) in the humerus for at least 1 time point after infection in 5 of 6 animals, with an average 2.2-fold increase (range, 1.6–3.2) in these 5 NHPs (Fig. 7; Supplemental Fig. 5). The increase was predominantly detected at the last 2 imaging time points. The determined SUV_mean_ of the bone marrow was lowest in 2 animals (Ji09002 and J09017), which exhibited the lowest CT score in lung lesions (Figs. 4 and 7). [^18^F]F-DPA714 showed only a mild increase in SUV_mean_ in the first 3 wk after infection, after which it decreased back to baseline values.

##### Spleen

In the spleen, we observed lower uptake of [^64^Cu]Cu-SIRPα-Nb before and after infection than of [^18^F]F-DPA714. However, in 4 of 6 investigated macaques, [^64^Cu]Cu-SIRPα-Nb uptake in the spleen was increased at the last imaging time point (day 51 after infection). As highlighted in Figure 7, we also detected a different [^64^Cu]Cu-SIRPα-Nb biodistribution pattern at this time point, suggesting an altered excretion pattern of [^64^Cu]Cu-SIRPα-Nb. In sharp contrast, [^18^F]F-DPA714 PET did not exhibit significant alterations of splenic uptake after SARS-CoV-2 infection (Fig. 7; Supplemental Fig. 6).

## DISCUSSION

After 4 y of pandemic, with more than 7 million deaths and ongoing health care and economic strain, SARS-CoV-2 is becoming a more controllable, seasonal disease (6*)*. However, emerging variants and long COVID remain major challenges. Deeper understanding of immune response and infection mechanisms is crucial for better patient care.

Here, we present for the first time, to our knowledge, insights into the spatiotemporal dynamics of myeloid cell–related inflammatory processes within the whole body of cynomolgus macaques during SARS-CoV-2 infection. Thus, we performed PET imaging with 2 tracers, [^64^Cu]Cu-SIRPα-Nb and [^18^F]F-DPA714, with different cellular targets. In line with our recently published results on [^18^F]F-DPA714 in a NHP SARS-CoV-2 infection model (11*)*, we determined increased PET uptake in the affected and unaffected lung tissue within the first days after SARS-CoV-2 infection, which was not the case for [^64^Cu]Cu-SIRPα-Nb. In contrast, only [^64^Cu]Cu-SIRPα-Nb PET revealed enhanced uptake patterns in the upper airway and the mediastinal LNs in the first scan at day 3 after infection. Although in this study we did not directly measure the number of myeloid cells present in the lung or other tissues over time, we have previously observed that the number of monocytes or macrophages in lung lavages increased on average 4-fold by day 2 and 10-fold by day 4 after SARS-CoV-2 infection (oral communication, Koopman 2024). Several groups indicate that TSPO is preferentially upregulated in antiinflammatory macrophages, which are subject of depletion during the inflammatory process associated with SARS-CoV-2 (24–26*)*. In contrast, SIRPα represents a broader pan-myeloid cell surface marker, including monocytes, macrophages, natural killer cells, neutrophils, and dendritic cells (15*)*. [^64^Cu]Cu-SIRPα-Nb also binds cSIRPγ, which is expressed, to a lower extent, on human and NHP T cells (27*)*. These findings may explain why different uptake patterns were seen and indicate that pan-myeloid cell tracers might be favorable for the visualization of proinflammatory processes compared with TSPO-targeting and other antiinflammatory macrophage–directed PET tracers (25*)*.

Moreover, we revealed enhanced [^64^Cu]Cu-SIRPα-Nb uptake in the bone marrow at the later imaging time points (24 and 52 d after infection) as a potential measure of chronic inflammatory processes, as described for long COVID (6*)*. The increased uptake patterns at later imaging time points may further indicate enhanced responsiveness of innate immune cells induced by certain infections and vaccines, known as trained immunity, that contributes to long-term inflammatory sequelae (28*)*. In line with this, a histopathologic examination of patients who died from COVID-19 revealed a significant higher fraction of CD11b-positive myeloid cells than in a noninfected control group (29*)*. Furthermore, analysis of the bone marrow of COVID-19 patients revealed hemophagocytic histiocytes as a sign for overwhelming inflammation (30*)*. Nevertheless, the extent to which these finding can help to identify severe and chronic disease development by imaging or bone marrow aspirations remains to be elucidated.

Limitations of our study are the lack of histologic verification of diseased NHPs, the limited number of subjects, and the missing disease variety. Even without this variety, there is still diversity in immune cell profiles in course of infection and determined imaging results. With this, it can be concluded that noninvasive imaging of macrophage migration dynamics during SARS-CoV-2 infection offers great potential in patients to possibly distinguish predisposal features, particularly when correlated to COVID-associated comorbidities and long COVID symptoms. In addition, PET imaging with a CD8-directed radiotracer revealed distinct T cell–derived uptake patterns in active and convalescent COVID-19 patients (31,32*)*. Furthermore, distinct PET uptake patterns were observed using [^18^F]FDG in the lungs, brain, or bone marrow of COVID-19 patients that correlated with disease severity or long COVID (33–35). Within this scope, imaging with dedicated PET tracers can be of additional value to current diagnostic approaches.

## CONCLUSION

The [^64^Cu]Cu-SIRPα-Nb biomarker investigated here represents a possibility for PET imaging of myeloid cells, which may contribute to better understanding of the pathophysiology of infection, the hyperinflammatory response in the active phase of the infection, and long-term inflammatory processes. The results support the development of both better diagnostic evaluation and macrophage-directed antiinflammatory therapeutic approaches.

## DISCLOSURE

This work was supported by funding from Transvac2 call 2211-15, Cluster of Excellence iFIT 2180 “Image Guided and Functionally Instructed Tumor Therapies,” University of Tuebingen, the Werner Siemens Foundation, and the European Virus Archive Global project, which has received funding from the European Union’s Horizon 2020 research and innovation program under grant agreement 87L029. Kinga Böszörményi was supported by the European Union’s Marie Skłodowska–Curie Innovative Training Network HONOURs (grant agreement 721367). Furthermore, the project was supported by the German Federal Ministry for Economic Affairs and Climate Action and the European Social Fund as part of the EXIST program (03EFVBW253–REVELICE). No other potential conflict of interest relevant to this article was reported.

## Notes

KEY POINTS **QUESTION:** What are the temporal dynamics of inflammatory processes in the body during SARS-CoV-2 infection, as investigated with a myeloid-directed PET tracer? **PERTINENT FINDINGS:** In vivo PET/CT revealed increased uptake of the SIRPα tracer in mediastinal LNs of all animals but not in lung lesions. In contrast, [^18^F]F-DPA714 showed increased uptake in both lung lesions and unaffected lung lesions, but the increase in uptake was less pronounced in mediastinal LNs. **IMPLICATIONS FOR PATIENT CARE:** The effects we observed in the body could have important clinical implications and need more in-depth analysis on the cell types involved. Specific molecular imaging adds to clinical and virologic assessments to be able to understand the pathophysiology and immune-phenotypic changes during SARS-CoV-2 infection, which in some cases can persist for months.
